# Supplementary material for: Blocking CCN2 Reduces Established Palmar Neuromuscular Fibrosis and Improves Function Following Repetitive Overuse Injury
Source: Int J Mol Sci. 2023 Sep 8;24(18):13866. doi: 10.3390/ijms241813866 (PMC10531056; doi:10.3390/ijms241813866)
Supplement: Supplementary file 1 [file ijms-24-13866-s001.zip › Supplemental Fig, legend, table.pdf]

Table S1. Fibrosis related protein levels (tested via ELISA) in forelimb muscles and associated connective tissues. Data previously graphed and reported in Barbe et al, 2020, FASEB [ref 34 of main text].

Analyte (pg/µg total protein)	Control (n=7-10)	HRHF-Untreated (n=6-9)	HRHF-Rest/IgG (n=5)	HRHF-Rest/anti-CCN2 (n=6)	ANOVA results
Collagen 1	1.35 ± 0.09	5.112 ± 0.49[aa]	4.63 ± 0.34[aa]	3.06 ± 0.26[a,bb,c]	p<0.0001
Collagen 3	0.05 ± 0.009	0.16 ± 0.009[aa]	0.21 ± 0.02[aa]	0.12 ± 0.02[a,c]	p<0.0001
CCN2[1]	0.02 ± 0.003	0.05 ± 0.005[aa]	0.05 ± 0.004[aa]	0.03 ± 0.003	p<0.0001
TGF-β[2]	0.02 ± 0.003	0.07 ± 0.01[aa]	0.08 ± 0.01[aa]	0.04 ± 0.006[b,c]	p<0.0001
FGF-2[3]	0.004 ± 0.0003	0.006 ± 0.001	0.006 ± 0.0004[aa]	0.005 ± 0.0001	p = 0.004

Means ± SEM shown; [a]:p<0.05 and [aa]:p<0.01, compared to controls; [b]:p<0.05 and [bb]:p<0.01, compared to HRHF-Untreated; [c]: p<0.05, compared to HRHF-Rest/IgG; [1]CCN2 = cell communication network factor 2; [2]TGF-β = transforming growth factor beta; [3]FGF-2; fibroblast growth factor 2.

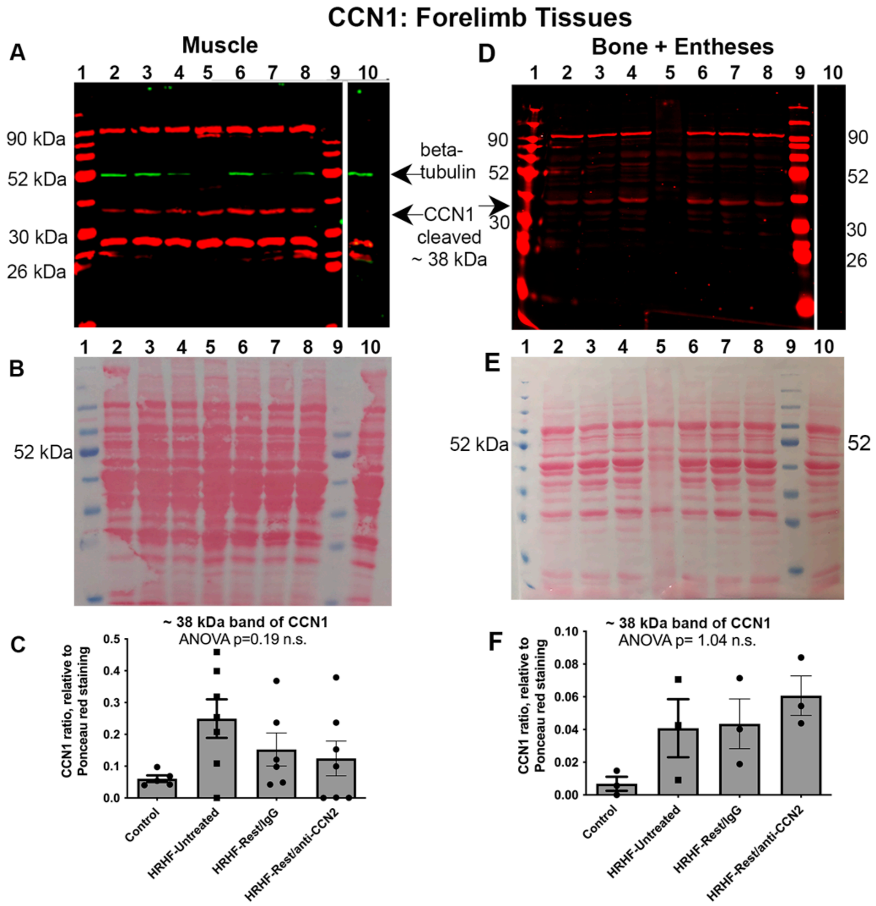

Figure S1. CCN1 protein levels in forelimb muscles and distal forelimb/wrist bone and entheses. A) Representative Western blot of forelimb muscles probed with anti-CCN1. Lanes loaded as: 1 = Ladder, 2 = HRHF-Untreated, 3 = HRHF-Rest/IgG, 4 = HRHF-Rest/anti-CCN2, 5 = Control, 6 = HRHF-Untreated, 7 = HRHF-Rest/IgG, 8 = HRHF-Rest/anti-CCN2, 9 = Ladder, 10 = secondary antibody only (no primary, not the two non specific bands at 30 and 28 kDa. Bands were detected at ~90 and ~38 kDa with the anti-CCN1 antibody; only the ~38 kDa band is a known band. This Western blot was also probed for beta tubulin, as shown in green at ~ 52 kDa. B) Matching Ponceau S red stained membrane, used to show protein loading levels. C) Quantification of the CCN1 ~ 38 kDa kDa band with expression shown as a ratio to the Ponceau S red staining in the matching lane. An ANOVA was performed with a Fisher LSD post hoc test.

Mean ± SEM is shown. D) Representative Western blot of distal forelimb/wrist bone and entheses (bones + etheses) probed with anti-CCN1. Lanes loaded the same as described for Panel A. Bands were detected at ~90 and ~38 kDa with the anti-CCN1 antibody; only the ~38 kDa band is a known band. E) Matching Ponceau S red stained membrane, used to show protein loading levels. F) Quantification of the CCN1 ~ 38 kDa kDa band with expression shown as a ratio to the Ponceau S red staining in the matching lane. An ANOVA was performed with a Fisher LSD post hoc test. Mean ± SEM is shown; n.s. = not significant.
